# Challenges Faced by Parents in Preventing Online Child Sexual Exploitation and Abuse: Protocol for a Systematic Review

**DOI:** 10.2196/80968

**Published:** 2026-02-09

**Authors:** Neelam Punjani, Wenting Yan, Lisa Hartling, Amber Hussain, Farah Elgaweesh, Shannon Scott

**Affiliations:** 1 College of Health Sciences, Faculty of Nursing, University of Alberta, 5-172 ECHA, 11405 87 Avenue NW, Edmonton, AB, T6G 1C9, Canada; 2 Pediatrics Department, Faculty of Medicine and Dentistry, University of Alberta, 2J2.00 Walter C. Mackenzie Health Sciences Centre, 8440 112 St. NWEdmonton, AB, T6G 2R7, Canada, 1 7806918938

**Keywords:** digital safety, child online sexual exploitation, parental perspectives, prevention barriers, systematic review

## Abstract

**Background:**

Online child sexual abuse and exploitation have become a global issue affecting the health and well-being of children and adolescents. Unlike traditional forms of sexual abuse and exploitation, online sexual abuse presents unique challenges due to the complexity of the situations involved. Parents play a crucial role as the first line of defense in preventing these incidents, yet the barriers they encounter have not been thoroughly explored.

**Objective:**

The objective of the systematic review is to identify the challenges parents face in preventing online child sexual abuse, to explore the factors that contribute to those challenges, and to highlight gaps in knowledge and practice regarding how to best support them.

**Methods:**

A comprehensive search will be conducted in MEDLINE (Ovid), Embase (Ovid), PsycINFO (Ovid), HealthSTAR (Ovid), Sociological Abstracts (ProQuest), CINAHL (EBSCOhost), Scopus (Elsevier), and the Cochrane Library (Wiley). The strategy will be developed with a research librarian. Regarding inclusion criteria, this review will include primary research studies (qualitative, quantitative, or mixed methods) that examine the perspectives of parents or primary caregivers of children and adolescents on online child sexual exploitation and abuse (OCSEA) prevention. Studies must focus on parental attitudes, fears, perceptions, or challenges related to OCSEA. Only studies published in English from 2018 onward will be considered. Studies that do not report on parental perspectives will be excluded. Two reviewers will independently screen studies and extract data, with conflicts resolved by a third reviewer. Findings will be narratively synthesized, following the PRISMA (Preferred Reporting Items for Systematic Reviews and Meta-Analyses) framework.

**Results:**

The search strategy has been developed in collaboration with a health sciences librarian and followed PRISMA-Search guidelines. Database searches, screening, and analysis are planned to commence in August, 2025. The findings of the review are anticipated to be ready by Febuary 2026 and will be presented following the PRISMA reporting standards.

**Conclusions:**

This protocol aims to structure a comprehensive and transparent process for a systematic review, which is expected to contribute to a better understanding of parental challenges in preventing OCSEA and provide insights into the types of supportive resources they need to combat OCSEA. This information will contribute to the development of comprehensive strategies that equip parents with the knowledge and tools necessary to effectively safeguard their children in the digital landscape.

## Introduction

Child sexual abuse (CSA) occurs when a child engages in sexual activity that they do not comprehend, cannot consent to, are unaware of, or are not prepared for [1]. It also includes illegal acts; for example, in Canada, 16 sexual offenses in the Criminal Code could apply to CSA [2]. CSA includes different types of abuse, such as sexual touching and sexual harassment, which can involve unwanted words, gestures, or physical contact of a sexual nature [3].

Child sexual exploitation (CSE) is a type of sexual abuse, with a distinguishing feature being the element of exchange [4,5]. This includes coercive situations in which a child or young person is pressured, manipulated, or tricked into sexual acts in exchange for something they may require or desire [6]. Many children are led to believe that they are in loving, consensual relationships, which can normalize these exploitative situations for them. This grooming process can obscure the reality of the exploitation taking place.

Both CSA and CSE represent severe forms of abuse. When they occur online or through information and communication technologies (ICTs), these abuses can collectively be referred to as online child sexual exploitation and abuse (OCSEA). As digital technology increasingly integrates into everyday life, OCSEA introduces new challenges for prevention and intervention in contemporary society.

OCSEA includes different types of sexual abuse or exploitation that occur online, including online solicitation, grooming, watching pornographic materials, forced or unwanted sexting, image-based abuse, sexual extortion, and exploitation [7-9]. Through ICTs, children may be exposed to cyberbullying, harmful or abusive content, and online grooming by potential predators, placing them at risk of exploitation and abuse [10]. Therefore, the development of technology has reshaped how CSA and CSE are perpetrated and examined, putting children at greater risk and increasing the extent of possible harm. Evidence suggests that when online abuse is categorized as sexual abuse, its incidence rates surge dramatically [11]. Thus, it is crucial to recognize OCSEA as a distinct concern. The breadth of this issue is extensive, and its impact on survivors is more severe than ever. The survivors of OCSEA may experience long-term effects, including problems in building relationships with other individuals in daily life, alienation from family and friends, mental illness, suicide attempts, self-harm, or even participating in criminal behaviors [12].

Although scholars acknowledge the seriousness of OCSEA, it remains an underreported problem for various reasons [13]. Currently, OCSEA is emerging as a significant global public health crisis. Statistics reveal that approximately 20% of children worldwide are affected by OCSEA [3]. Different countries have reported their prevalence. For example, Finkelhor et al [7] analyzed the prevalence of online CSA in the United States and determined that online CSA prevalence was 15.6%. Walsh et al [14] reported that in Australia, the percentage of adults soliciting sex online was 17.7%, and the percentage of nonconsensual dissemination of sexual images of children was 7.6%. In Canada, the percentage of CSE online rose by 18% from 2021 to 2022, with child pornography being the most prevalent offense [15]. The COVID-19 pandemic exacerbated the situation, as lockdowns confined children at home, leading to a shift to online learning environments, thereby increasing exposure to OCSEA. Meanwhile, the issue is also latent; studies show that approximately 1 in every 3 children does not disclose their abuse to anyone, indicating that sexual offenses against children are likely underreported and suggesting that actual incidence rates are much higher than currently reflected [15,16].

To protect future generations, it is essential to proactively address and prevent OCSEA. However, a significant number of children or even their parents are unaware of what OCSEA entails. Therefore, we must educate and empower children and their parents on this issue, enabling them to recognize and report incidents of OCSEA, thus breaking the cycle of abuse [13].

Research has consistently highlighted the critical role that parents play in safeguarding their children from CSA [17-19]. According to Rudolph et al [18], parents serve as a protective factor both directly and indirectly. Directly, they provide supervision, monitor their children’s activities, and engage in their daily lives, thereby offering a shield against potential risks. Indirectly, parents foster their children’s general self-efficacy, competence, well-being, and self-esteem, which are vital for resilience. As the primary caregivers, parents know their children best and are attuned to their emotional changes. Their involvement is the most effective way to create a secure environment where children feel safe and protected [18]. However, challenges persist regarding effective parental involvement [20]. Key questions arise: How can parents convey a comprehensive prevention message? How can they bridge knowledge and communication effectively with their children? The context of OCSEA, compounded by the rise of ICTs, introduces new barriers for parents as protectors. Many parents may lack the necessary knowledge or skills to guide their children through the growing array of online risks and supervise their internet use effectively. Additionally, detecting OCSEA has become increasingly complex; thus, traditional methods of warning children about threats may no longer suffice. A broader approach is essential—one that empowers parents to guide their children in managing risks and enhancing their capacity to protect themselves from harm.

Several studies have examined parental preventative practices regarding traditional forms of CSA and CSE [17,21,22]. However, there has been less focus on the unique challenges parents face in preventing OCSEA. Novianti [23] investigated and identified several vulnerability factors contributing to OCSEA, highlighting the lack of parental supervision in children’s internet use as a significant concern. Despite this, few studies have explored the barriers and challenges parents encounter when trying to mitigate this vulnerability. Addressing these issues is crucial for developing effective programs and strategies that meet the needs of the digital age. Therefore, a systematic review will be undertaken to identify previous research that has examined the challenges parents face and the factors that contribute to those challenges in preventing OCSEA. This review will illuminate gaps in current knowledge and practice regarding how to best equip parents with the necessary information and skills. Ultimately, this research will provide evidence-informed guidance for interventions, policymaking, and future research to enhance the prevention of OCSEA.

This systematic review will be guided by the following research questions:

- What types of evidence exist regarding the challenges parents face in preventing OCSEA?
- How have parents’ perceptions related to OCSEA been described in the existing literature?
- What gaps exist in the current evidence base regarding parental involvement in preventing OCSEA?

## Methods

This review will be conducted following the PRISMA (Preferred Reporting Items for Systematic Reviews and Meta-Analyses) statement [24].

### Identifying Relevant Studies

To identify the relevant studies*,* a systematic literature search will be conducted by an experienced health sciences librarian. Multimedia Appendix 1 shows the proposed search strategy to be used to search MEDLINE. Searches will be performed in the following databases: MEDLINE, Embase, PsycINFO, HealthSTAR, Sociological Abstracts via ProQuest, CINAHL via EBSCOhost, Scopus via Elsevier, and the Cochrane Library via Wiley. These databases will be searched using a combination of natural language vocabulary and controlled terms (eg, Medical Subject Headings [MeSH]), where available. Natural language terms will be derived from three main concepts: (1) online CSE, online CSA, or technology-based CSA; (2) children and adolescents; and (3) parents or caregivers. Other search methods will also be used to retrieve additional evidence. These methods will include hand searches of the reference lists and forward citation searches.

Inclusion and exclusion criteria were established using the population, concept, context (PCC) framework (Textbox 1). The PCC framework is recommended by the Joanna Briggs Institute to identify the main concepts in the primary review questions [25]. Articles will be included if the study examines parents’ perceptions, attitudes, fears, and challenges related to the prevention of OCSEA among their children and adolescents who are younger than 18 years. Articles published in English from 2018 to date will be included to capture the most current evidence following key global developments in online child protection, including the adoption of the WeProtect Global Alliance’s Model National Response and United Nations Children's Fund’s (UNICEF) call for coordinated action in child online protection [26,27]. Meanwhile, since 2018, the digital environment has experienced substantial qualitative change, with platforms such as Instagram and TikTok adopting algorithm-driven technologies that enable more complex engagement patterns and richer interactive features, while reaching over one billion users globally [28,29]. In addition, this time frame also includes the COVID-19 pandemic period, capturing both prepandemic- and pandemic-related shifts in related challenges. All primary research studies, irrespective of study design, will be considered. In terms of gray literature, we do not intend to include a targeted search of gray literature sources. However, student theses or dissertations identified during the database search and full-text screening process will be screened and included using the same eligibility criteria and will be documented alongside other included records.

Textbox 1. **Inclusion and exclusion criteria.**

**Inclusion criteria**

- Parents, caregivers, and guardians of adolescents and children younger than 18 years
- Studies that explore challenges faced by parents in preventing online child sexual exploitation and abuse
- Online or digital environments across various platforms (eg, social media and messaging apps)
- Any country or cultural context
- All primary research studies, including qualitative, quantitative, and mixed methods study designs
- Published research, including peer-reviewed journal articles and gray literature such as student dissertations and theses, provided they report original research findings relevant to the review questions
- Articles published from 2018 onward
- Publications in English

**Exclusion criteria**

- Studies that do not involve parents, caregivers, and guardians of adolescents and children younger than 18 years
- Studies that do not mention parental perceptions, attitudes, fears, or challenges related to online child sexual exploitation and abuse
- Studies focused on law enforcement, online safety, educators, or other professionals without parental involvement
- Studies involving adult survivors of online sexual exploitation
- Studies that do not focus on the prevention aspect of online child sexual exploitation and abuse
- Studies that focus exclusively on children or adolescents younger than 18 years without any exploration of parental challenges or roles
- Studies mentioning parental perceptions, attitudes, fears, or challenges related to child sexual exploitation or child sexual abuse or any other nononline format but not online child sexual exploitation and abuse
- Secondary research, such as reviews and content analysis, commentary, and other
- Opinion pieces, blog posts, or editorials without empirical evidence
- Published studies or gray literature that do not report original research findings
- Articles published before 2018
- Non-English publications

### Study Selection

Covidence is a web-based tool for systematic review management that offers a platform for importing article records and conducting independent screening. In this study, after exporting all articles from databases, Covidence will be used to import all articles. Duplicates will be detected and removed automatically in Covidence, facilitating the title and abstract screening and full-text screening phases.

A total of 3 reviewers will be involved in the screening process. Two reviewers will independently screen titles and abstracts, categorizing them as included, excluded, or unclear. Studies marked as included or unclear by either reviewer will proceed to full-text screening. Disagreements at any stage will be resolved through discussion or by a third reviewer. This approach ensures a high level of reliability and consistency in the selection of eligible studies. Additionally, eligible studies will also be identified by manually screening the reference lists of related studies. After determining all eligible studies, a diagram demonstrating the flow of the screening process will be produced and reported in a PRISMA flow diagram (see Multimedia Appendix 2).

### Data Extraction and Critical Appraisal

A data extraction sheet has been created by researchers on the team and will be used to extract data from all studies that meet the inclusion criteria. After the initial version of the data extraction sheet is created, 2 reviewers will select 10% (2/20) of the total number of included studies to pilot the sheet [30]. They will independently complete data extraction for these 2 studies using the draft sheet to assess its comprehensiveness. If any issues arise during the piloting process, all members of the research team will discuss how to revise and improve the data extraction sheet accordingly before proceeding with the full data extraction. Following the pilot extraction, the research team will compare the results, identify any inconsistencies, and revise the extraction sheet to produce the final version.

Data extracted from each study will include bibliographic details, study design, study population, identified challenges, factors contributing to the challenges, conclusions, research implications, limitations, and identified research gaps. The bibliographic details will include the record number in Covidence, authors, titles, publication year, and the country or region where the study was conducted. The study design will specify whether the study is qualitative (interviews, focus groups, etc), quantitative (surveys, questionnaires, etc), or mixed methods. It will also indicate the type of OCSEA discussed and the study’s research question or hypothesis. The study population will include participant information, such as sample size, age distribution, gender distribution, marital status, ethnic background, and religion. The findings will highlight the challenges identified in the selected studies, including the influencing factors that contributed to these challenges. Two reviewers will independently extract the data, which will be compared for consistency; discrepancies will be identified and resolved through discussion. If there is any uncertainty about the included papers or missing data, the authors will be contacted for clarification.

The methodological quality and potential risk of bias of included studies will be assessed using the Joanna Briggs Institute (JBI) Critical Appraisal Tools by two independent reviewers [31]. Any discrepancies will be resolved through discussion as well. As a single JBI checklist cannot cover all study designs, we will combine relevant JBI checklists for different study designs into a single appraisal framework, with minor adaptations as needed. Study quality will be categorized as high (≥75% of criteria met), moderate (50%-74%), or low (<50%).

### Data Analysis and Presentation

The findings of the study will be presented narratively using the PRISMA checklist. A tailored approach, a descriptive numerical summary, and a thematic analysis will be used to synthesize the data and create an evidence table that will summarize key findings from each study [32]. This will include each study’s information, including goals or objectives, population, methods, and outcomes of interest. Next, the data will be aggregated and analyzed thematically according to the study’s outcomes. A descriptive (narrative) analysis will be conducted to identify common themes in the challenges faced by parents in providing information about OCSEA to their children.

## Results

The search strategy has been finalized with input from a health sciences librarian. Database searches and gray literature screening are scheduled to begin in August 2025. Results from the review are expected by Febuary 2026 and will be reported in accordance with the PRISMA guidelines, and search methods will be reported following PRISMA-Search guidance. This protocol is structured to address PRISMA-recommended protocol elements, with detailed search strategies and supporting materials provided in the appendices.

## Discussion

### Overview

OCSEA is a growing global concern, with children increasingly at risk in the digital environment. While the role of parents as protectors has been acknowledged in the literature addressing traditional forms of CSA and CSE, less is known about the distinct challenges they face in addressing OCSEA. The unique nature of online abuse, such as often invisible, technology-mediated, and rapidly evolving, creates specific barriers for parental involvement in prevention efforts. Despite the critical importance of equipping parents to safeguard their children online, research on their lived experiences, perceived barriers, and informational needs remains fragmented.

We anticipate that this review will map different categories and characteristics of parental challenges related to OCSEA using an established methodological framework and a comprehensive search strategy. We expect that the findings will highlight the complexity of parental roles within contemporary digital environments and provide valuable insight into the areas where parents feel unsupported or underprepared, as well as gaps in the available evidence that could inform future targeted interventions.

By highlighting the nature and extent of these challenges, this review has the potential to guide the development of educational resources, public health messaging, and policy reforms aimed at empowering parents. It also aims to support interdisciplinary collaboration among researchers, educators, child protection specialists, and policymakers to foster safer digital environments for children. The findings will contribute meaningfully to the understanding of OCSEA prevention and inform future research and interventions designed to enhance parental capacity in online safety and abuse prevention.

### Limitations

The restriction to studies published from 2018 onward and the absence of a targeted gray literature search may result in the omission of earlier foundational work or unpublished evidence.

### Dissemination Plan

Results from this review will be disseminated through publication in a peer-reviewed journal and presentation at relevant academic and professional conferences. Findings may also be shared with stakeholders through knowledge translation activities.

## Supplementary material

10.2196/80968 Multimedia Appendix 1 MEDLINE search strategy.

10.2196/80968 Multimedia Appendix 2 PRISMA (Preferred Reporting Items for Systematic Reviews and Meta-Analyses) flow diagram.

## Abbreviations

CSA: child sexual abuse
CSE: child sexual exploitation
ICT: information and communication technology
MeSH: Medical Subject Headings
OCSEA: online child sexual exploitation and abuse
PCC: population, concept, context
PRISMA: Preferred Reporting Items for Systematic Reviews and Meta-Analyses
UNICEF: United Nations Children’s Fund
