# Supplementary material for: Challenges Faced by Parents in Preventing Online Child Sexual Exploitation and Abuse: Protocol for a Systematic Review
Source: JMIR Res Protoc. 2026 Feb 9;15:e80968. doi: 10.2196/80968 (PMC12890777; doi:10.2196/80968)
Supplement: Multimedia Appendix 1 [file resprot-v15-e80968-s001.docx]

**Appendix 1:** MEDLINE Search Strategy

| Ovid MEDLINE(R) ALL |  |
| --- | --- |
|  |  |
| 1 | Child Abuse, Sexual/ |
| 2 | Pedophilia/ |
| 3 | ((child* or adolescen* or teen* or youth* or young* or minor? or underage* or toddler* or infant* or baby or babies or school*) adj3 (exploit* or extort* or "sexual* abus*" or "sexual* manipulat*" or "sexual offen*" or "sexual* assault*")).mp. |
| 4 | P?edophil*.mp. |
| 5 | 1 or 2 or 3 or 4 |
| 6 | erotica/ |
| 7 | (porn or pornograph* or erotic*).mp. |
| 8 | ((sex* adj3 (text* or chat* or talk* or correspond* or contact* or interest*)) or sext*).mp. |
| 9 | Rape/ |
| 10 | (rape? or raping or rapist*).mp. |
| 11 | Sex Work/ |
| 12 | (prostitut* or "sex work*").mp. |
| 13 | ((sexual* or explicit) adj3 (photo* or image* or video* or material* or content*)).mp. |
| 14 | (nude? or nudity).mp. |
| 15 | sex* act*.mp. |
| 16 | ("sexual violence" or "sexual victim*").mp. |
| 17 | (cybersex* or "cyber sex*").mp. |
| 18 | or/6-17 |
| 19 | adolescent/ or exp child/ or infant/ or child, abandoned/ or child, adopted/ or child, exceptional/ or "child of impaired parents"/ or child, foster/ or child, orphaned/ or child, unwanted/ or minors/ or students/ |
| 20 | (child* or adolescen* or teen* or youth* or young* or minor? or underage* or toddler* or infant* or baby or babies or school*).mp. |
| 21 | 19 or 20 |
| 22 | 18 and 21 |
| 23 | 5 or 22 |
| 24 | internet/ or social media/ |
| 25 | (online* or internet* or web* or "social media" or facebook or twitter or instagram or "tik tok" or tiktok or tumblr or reddit or twitch or youtube or whatsapp or wechat or snapchat* or pinterest or quora or discord or mastodon or technolog* or digital* or virtual*).mp. |
| 26 | computers/ or computers, handheld/ or smartphone/ |
| 27 | (computer* or laptop* or ipad* or "smart phone*" or smartphone* or iphone* or android* or samsung).mp. |
| 28 | exp Cell Phone/ |
| 29 | ("cell phone*" or "text messag*" or email* or e-mail*).mp. |
| 30 | or/24-29 |
| 31 | exp parents/ or exp fathers/ |
| 32 | (parent* or father* or mother* or mom? or mum? or dad? or caregiver* or "care giver*" or guardian*).mp. |
| 33 | 31 or 32 |
| 34 | 23 and 30 and 33 |
| 35 | limit 34 to (english language and yr="2018 -Current") |
