# Supplementary material for: Challenges Faced by Parents in Preventing Online Child Sexual Exploitation and Abuse: Protocol for a Systematic Review
Source: JMIR Res Protoc. 2026 Feb 9;15:e80968. doi: 10.2196/80968 (PMC12890777; doi:10.2196/80968)
Supplement: Multimedia Appendix 2 [file resprot-v15-e80968-s002.docx]

**Multimedia Appendix 2:** PRISMA Flow Diagram (adapted from Source: Page MJ, et al. BMJ 2021;372:n71. doi: 10.1136/bmj.n71.)

**Identification of studies via databases and registers**

Records removed before screening:

Duplicate records removed by Covidence (n=)

Duplicate records removed manually (n=)

Records identified from:

Databases (n =)

Scopus (n =)

Embase (n=)

MEDLINE (n=)

PsycINFO (n=)

CINAHL (n=)

CENTRAL (n=)

**Identification**

Records screened (n=)

Records excluded from title and abstract screening (n=)

Reports excluded from full-text screening (n=):

Wrong study concept (n=)

Wrong context (n=)

Wrong study design (n=)

Cannot access the paper (n=)

Non-English paper (n=)

Wrong participant (n=)

Wrong publication year (n=)

**Screening**

Full-text records assessed for eligibility (n=)

Studies included in the review

(n=)

**Included**
